# Global trends and future predictions of pancreatic cancer incidence and mortality from 1990 to 2030: A comparative analysis of China, Korea, Japan, and the USA

**DOI:** 10.1371/journal.pone.0337181

**Published:** 2025-12-11

**Authors:** Kun Huang, Xiang Lan, Zhu Chen, Heng Xiao, Shunhu Jia, Chenyou Du

**Affiliations:** 1 Department of General Surgery, Mian Yang Hospital of Traditional Chinese Medicine, Mianyang, China; 2 Department of Hepatobiliary Surgery, The First Affiliated Hospital of Chongqing Medical University, Chongqing, China; Baylor Scott & White Research Institute, UNITED STATES OF AMERICA

## Abstract

**Background:**

Pancreatic cancer is a leading cause of cancer-related deaths globally, with rapidly increasing incidence and mortality rates in several regions. This study provides a comparative analysis of pancreatic cancer trends in China, Korea, Japan, and the USA, and projects these trends through 2030.

**Methods:**

Data from the Global Burden of Disease (GBD) study were used to examine trends in age-standardized incidence rates (ASIR), age-standardized mortality rates (ASMR), and disability-adjusted life years (DALYs) from 1990 to 2020. Joinpoint regression and age-period cohort (APC) models were employed to assess temporal trends. Future predictions of ASIR, ASMR, and DALYs were generated using predictive modeling to extend the analysis to 2030.

**Results:**

The study found that Japan and the USA have the highest incidence and mortality rates for pancreatic cancer, with predicted ASIR values expected to exceed 15 per 100,000 in Japan by 2030. Mortality trends follow a similar pattern, with males consistently showing higher ASMR than females across all regions. China is expected to see continued growth in DALYs, particularly among males, while Korea exhibits stable or slightly declining trends. The longitudinal age curves indicate that pancreatic cancer rates rise significantly with age, especially in individuals over 65 years. By 2030, Japan and the USA are projected to experience the greatest increase in pancreatic cancer burden, while Korea’s trends remain relatively stable.

**Conclusions:**

Pancreatic cancer continues to pose a significant public health challenge, particularly in aging populations. The predicted rise in incidence, mortality, and DALYs in Japan and the USA calls for urgent implementation of targeted screening and prevention strategies, particularly for older age groups and high-risk populations.

## Introduction

Pancreatic cancer, despite its relatively lower incidence compared to other malignancies, is one of the most lethal forms of cancer globally due to its poor prognosis and limited treatment options [1]. In recent years, advancements in early detection and treatment modalities have shown improvement in survival rates for various types of cancer; however, pancreatic cancer remains a major challenge in oncology [2]. Among the contributing factors to its high mortality rate are the late-stage diagnosis, the aggressive nature of the disease, and the unique tumor microenvironment, which is marked by desmoplasia and significant immune evasion mechanisms [3]. Consequently, there is an urgent need for more comprehensive analyses and innovative treatment strategies to reduce the burden of this disease [4–6].

Several epidemiological studies have highlighted disparities in the incidence and mortality trends of pancreatic cancer across different regions. For instance, while countries such as Japan and the United States have seen modest declines in mortality due to improved healthcare systems and early intervention, the burden of pancreatic cancer in China and Korea continues to rise [7–10]. This phenomenon is largely attributed to population aging, lifestyle changes, and genetic predispositions specific to these regions [11–13]. The Global Burden of Disease (GBD) study offers a valuable tool for tracking such epidemiological trends, providing insights into the distribution of pancreatic cancer and its associated risk factors on a global scale [14,15]. By leveraging GBD data, researchers can identify patterns that inform healthcare policies and targeted interventions for high-risk populations.

The countries selected for this study—China, South Korea, Japan, and the United States—were chosen for their diverse healthcare systems, socioeconomic conditions, and public health policies. These countries represent both high-income and middle-income economies across East Asia and North USA, allowing us to compare how variations in health policies, medical access, and lifestyle factors influence pancreatic cancer incidence and mortality. The healthcare systems of these countries vary significantly, with China undergoing rapid healthcare expansion, South Korea and Japan having established systems with strong cancer screening initiatives, and the United States grappling with healthcare access issues, especially among lower-income populations. Moreover, differences in dietary habits, such as higher consumption of salt and fermented foods in East Asia and rising obesity rates in the United States, further influence cancer trends.

The present study aims to conduct a comparative analysis of pancreatic cancer incidence and mortality in China, Korea, Japan, and the USA from 1990 to 2020, utilizing the GBD database. This analysis is crucial to understanding regional variations and the effectiveness of current preventive and therapeutic strategies in different healthcare contexts. By exploring these trends, we hope to contribute to the growing body of literature on pancreatic cancer and provide a foundation for future research efforts aimed at mitigating its global impact.

## Materials and methods

### Data source

This study utilized data from the Global Burden of Disease (GBD) 2021 study, which provides standardized global estimates of disease burden. We extracted data related to pancreatic cancer, including incidence, mortality, prevalence, and disability-adjusted life years (DALYs) for China, Korea, Japan, and the United States over the period 1990–2021. The GBD dataset is publicly available through the Institute for Health Metrics and Evaluation (IHME) and can be accessed at https://vizhub.healthdata.org/gbd-results/.

The definitions of the extracted indicators are as follows:

**Incidence**: The number of new pancreatic cancer cases per 100,000 population.

**Mortality**: The number of deaths attributed to pancreatic cancer per 100,000 population.

**Prevalence**: The total number of pancreatic cancer cases per 100,000 at a given time.

**DALYs**: A measure combining years of life lost (YLL) due to premature death and years lived with disability (YLD), reflecting both fatal and non-fatal disease outcomes.

Data were **stratified by age, sex, country, and year**, and downloaded using the GBD Results Tool with the following parameters:

**Cause**: Pancreatic cancer;

**Measure**: Incidence, Mortality, Prevalence, DALYs;

**Metric**: Number and Rate;

**Location**: China, Korea, Japan, and the United States;

**Sex**: Both;

**Age group**: All ages;

**Years**: 1990–2021

All data extraction and cleaning procedures were performed using R software (version 4.3.3). The ggplot2 and dplyr packages were used for data wrangling and graphical visualization.

### Ethical statement

This study used aggregated, de-identified, and publicly available data obtained from the Global Burden of Disease (GBD) 2021 Results Tool (Institute for Health Metrics and Evaluation: https://vizhub.healthdata.org/gbd-results/). No human participants or animal subjects were directly involved; therefore, approval from an institutional review board and informed consent were not required. If future research involves individual-level patient data, ethical approval will be obtained from the Institutional Review Board of Mianyang Hospital of Traditional Chinese Medicine, in accordance with the Declaration of Helsinki and applicable regulations.

### Statistical analysis

#### Joinpoint regression analysis.

Joinpoint regression was used to identify significant changes in trends of pancreatic cancer incidence and mortality. The **Joinpoint Regression Program (version 5.1.0.0)** was applied to calculate the **annual percent change** and detect joinpoints, which represent statistically significant changes in the linear trend over time.

For each country and gender, models were fitted separately. The regression starts with a straight line (0 joinpoints) and tests whether more joinpoints improve model fit. The annual percent change was calculated for each identified time segment, with a 95% confidence interval used to determine significance. This analysis allowed for identifying periods of rapid increase or decrease in pancreatic cancer burden [16].

### Age–period–cohort (APC) analysis

An **APC analysis** was performed to assess how age, period, and birth cohort influenced trends in pancreatic cancer incidence and mortality. This method distinguishes the effects of aging, specific periods (e.g., medical advancements, public health interventions), and generational changes in lifestyle or exposures.

The APC models were constructed using the **Epi package** in R, and the **relative risk (RR)** was estimated for each age group, period, and cohort. Longitudinal age curves were generated to visualize age-related risks, and period and cohort effects were plotted to understand time-specific and generational influences on pancreatic cancer trends.

### Future projections

Using the historical trends from 1990 to 2021, future projections for age-standardized incidence rates (ASIR), age-standardized mortality rates (ASMR), and **DALYs** for pancreatic cancer were generated for the period 2021–2030. Projections were modeled using **R software (version 4.3.3)**, with the **forecast** package. The predictive model assumed that current trends in demographics and healthcare access would continue, and gender-specific projections were generated for each country.

## Results

### The baseline characteristics of pancreatic cancer from 1990 to 2021 reveal significant variations in prevalence, incidence, deaths, and DALYs across different regions

Globally, the prevalence and incidence rates increased by 16.5% and 8.9%, respectively, while the death rate surged by 67.9%, indicating that despite advances in healthcare, pancreatic cancer remains a significant cause of mortality. The overall DALYs showed a modest 0.8% increase, reflecting a persistent global burden of disease. In China, prevalence and incidence rates showed substantial increases of 27.3% and 24.2%, respectively, with mortality rising dramatically by 112.8%, suggesting a worsening situation for pancreatic cancer in the country. DALYs in China increased by 70.5%, underscoring the growing impact of the disease. In contrast, while the USA had a higher prevalence and incidence compared to global averages (9.5 and 10.3 per 100,000), mortality decreased by 15.9%, and DALYs dropped by 23.7%, indicating improvements in cancer management and patient survival.

Korea and Japan also displayed distinct trends. In Korea, the prevalence increased by 25.2%, but the incidence remained stable. Mortality and DALYs decreased significantly by 32.9% and 32.8%, respectively, suggesting enhanced treatment and early detection efforts. Japan showed the highest prevalence (12.3 per 100,000) and a notable increase in incidence by 21.3%. However, mortality decreased by 33.6%, highlighting successful healthcare interventions, although DALYs increased slightly by 9%. These trends suggest that while incidence rates of pancreatic cancer are on the rise globally, regions like Korea, Japan, and the USA have made significant strides in reducing mortality and the overall disease burden, in contrast to China, where the burden continues to grow sharply (Table 1).

**Table 1:** Baseline characteristics of pancreatic cancer incidence, prevalence, mortality, and DALYs in China, Korea, Japan, and the USA (1990–2021).

	Prevalence (95% UI)			Incidence (95% UI)			Deaths (95% UI)			DALYs (95% UI)		
Location	no.	ASRs/100000	Percentage change in ASRs from 1990 to 2021	no.	ASRs/100000	Percentage change in ASRs from 1990 to 2021	no.	ASRs/100000	Percentage change in ASRs from 1990 to 2021	no.	ASRs/100000	Percentage change in ASRs from 1990 to 2021
Global	439001 (401739–471000)	5.1 (4.7 to 5.5)	16.5 (12.3 to 19.6)	508533 (462091–547208)	6 (5.4 to 6.4)	8.9 (4.5 to 12)	505752 (461224–543899)	5.9 (5.4 to 6.4)	67.9 (63.9 to 67.9)	11316963 (10464697–12169336)	130.3 (120.5 to 140.1)	0.8 (−2 to 3.1)
China	95524 (75563–116662)	4.5 (3.6 to 5.5)	27.3 (20.3 to 32.9)	118665 (94623–144663)	5.6 (4.5 to 6.8)	24.2 (17.6 to 29.4)	119602 (95654–145218)	5.7 (4.6 to 6.9)	112.8 (121.5 to 95.5)	2930317 (2301049–3575079)	137.2 (108.1 to 166.7)	70.5 (92.4 to 51.7)
USA	54303 (50464–56689)	9.5 (8.9 to 9.9)	17 (15.3 to 18.3)	61340 (56243–64259)	10.3 (9.5 to 10.8)	10.7 (8.7 to 11.9)	57098 (52182–59928)	9.5 (8.7 to 9.9)	−15.9 (−29.8 to −1.2)	1192434 (1124834–1238029)	210 (199.5 to 217.4)	−23.7 (−36.4 to −9.7)
Korea	7552 (5988–9197)	8 (6.4 to 9.8)	25.2 (17 to 31.9)	7784 (6114–9429)	8.2 (6.5 to 10)	0 (−7.6 to 4.9)	7101 (5596–8633)	7.5 (5.9 to 9.1)	−32.9 (−36.8 to −30.4)	146129 (116596–177361)	155.4 (124.3 to 188.7)	−32.8 (−38.1 to −29)
Japan	47434 (39193–52343)	12.3 (10.6 to 13.2)	36.6 (25.6 to 40.9)	46502 (39057–50709)	11.5 (10.1 to 12.3)	21.3 (13.6 to 25.1)	42065 (35527–45867)	10.3 (9.1 to 11)	−33.6 (−35.7 to −31)	709065 (625627–755944)	215.3 (196 to 225.7)	9 (4 to 11.2)

### Trends in prevalence and incidence rates of pancreatic cancer by gender and region from 1990 to 2021

**Fig 1:**
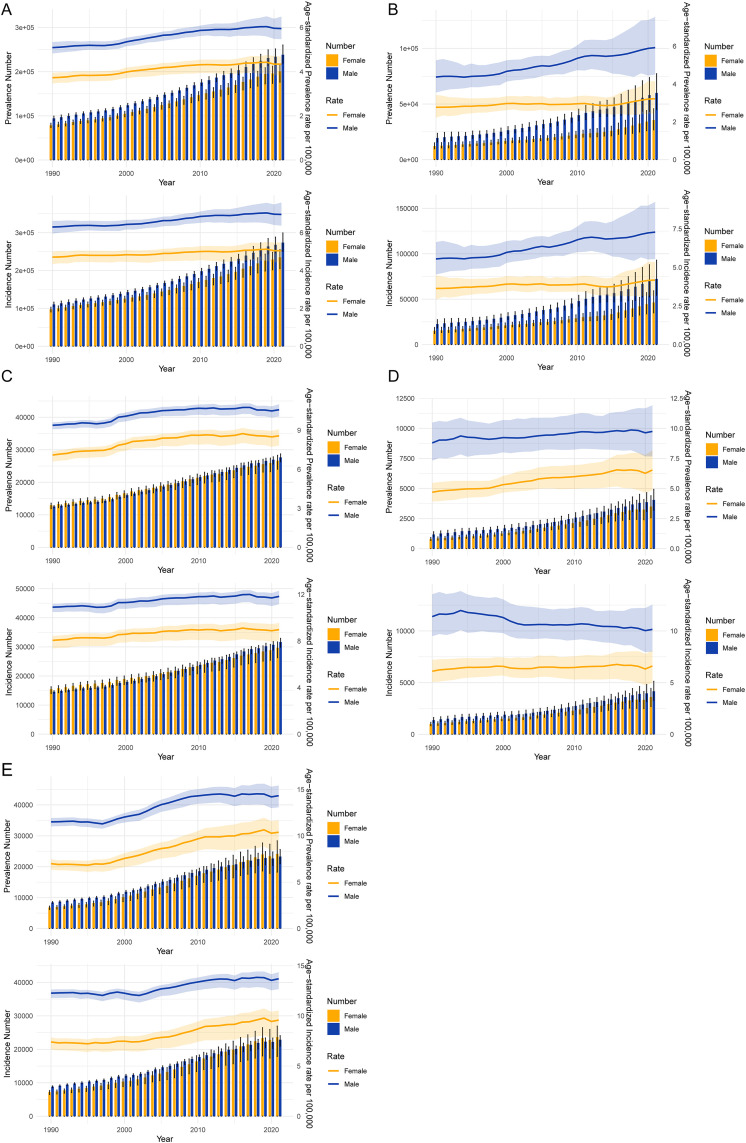
Trends in Prevalence and Incidence Rates of Pancreatic Cancer by Gender and Region (1990–2021). (A) **Global trends**: Both prevalence and incidence have steadily increased over time, with men showing higher age-standardized rates (ASRs) than women. (B) **China**: A rising trend in both prevalence and incidence is observed, with men showing substantially higher rates throughout the study period. (C) **USA**: Prevalence and incidence have also increased notably, with a more pronounced rise among men, although the gender gap is smaller compared to China. (D) **Korea**: Prevalence rates have increased, but incidence remains relatively stable, with a slight male predominance. (E) **Japan**: Prevalence and incidence continue to rise for both genders, with men consistently having higher rates compared to women.

Fig 1 illustrates the temporal trends in the prevalence and incidence of pancreatic cancer by gender (male and female) across five regions: global, China, USA, Korea, and Japan, from 1990 to 2021. **Panel A** presents global data, showing a steady increase in both prevalence and incidence for both genders. Men consistently show higher age-standardized rates (ASRs) than women across the study period. **Panel B** focuses on China, where a rising trend in both prevalence and incidence is observed, with men showing substantially higher rates than women throughout. **Panel C**, representing the USA, reveals a notable increase in prevalence and incidence, with a more pronounced rise in men, although the gap between genders is smaller compared to China. **Panel D** displays data from Korea, showing a stable incidence rate over time, with a slightly higher prevalence in men. Finally, **Panel E** for Japan indicates a continuous increase in prevalence and incidence for both genders, with men consistently having higher rates.

### Age and gender distribution of pancreatic cancer prevalence and incidence in different regions

**Fig 2:**
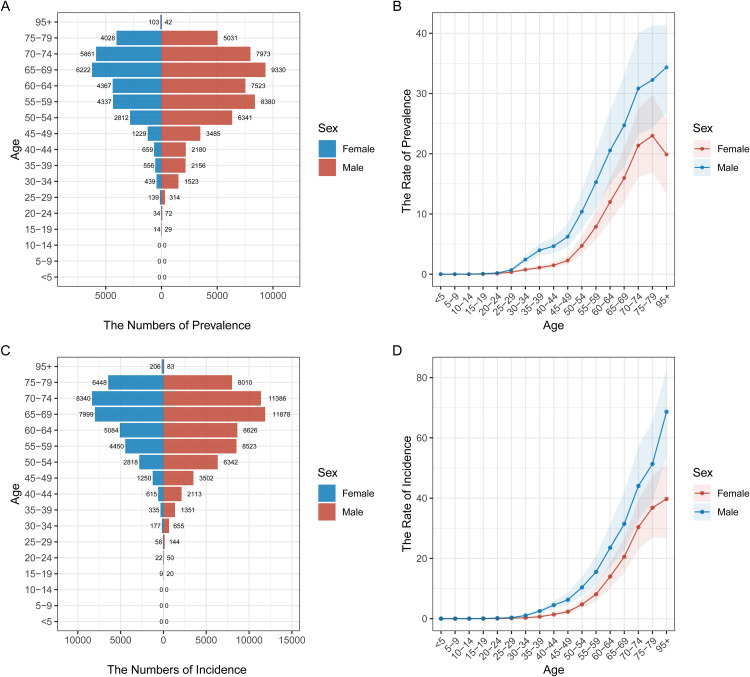
Age and Gender Distribution of Pancreatic Cancer Prevalence and Incidence in China (1990–2021). (A) **Prevalence by age and gender**: The number of prevalent cases is higher in males across all age groups, with significant increases among those aged 75 and older. (B) **Rate of prevalence by age group**: Prevalence rates rise steadily with age, with males consistently having higher rates than females, particularly in older age groups. (C) **Incidence by age and gender**: The number of new pancreatic cancer cases per age group is higher in males, with a marked increase in older individuals (65 + years). (D) **Rate of incidence by age group**: Incidence rates show similar trends, increasing significantly with age and with males being disproportionately affected.

**Fig 3:**
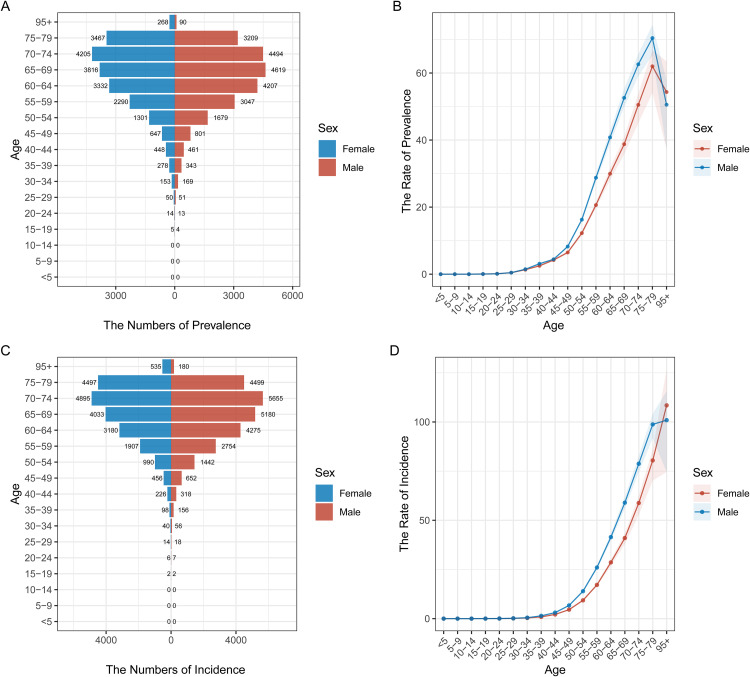
Age and Gender Distribution of Pancreatic Cancer Prevalence and Incidence in the USA (1990–2021). (A) **Prevalence by age and gender**: Males have a higher prevalence of pancreatic cancer compared to females, especially in the older age groups (70 + years). (B) **Rate of prevalence by age group**: The prevalence rate increases sharply with age for both genders, with men consistently showing higher values. (C) **Incidence by age and gender**: Males show higher numbers of new cases, particularly among individuals over 70 years. (D) **Rate of incidence by age group**: Incidence rates escalate with age, with men showing higher rates across most age groups.

**Fig 4:**
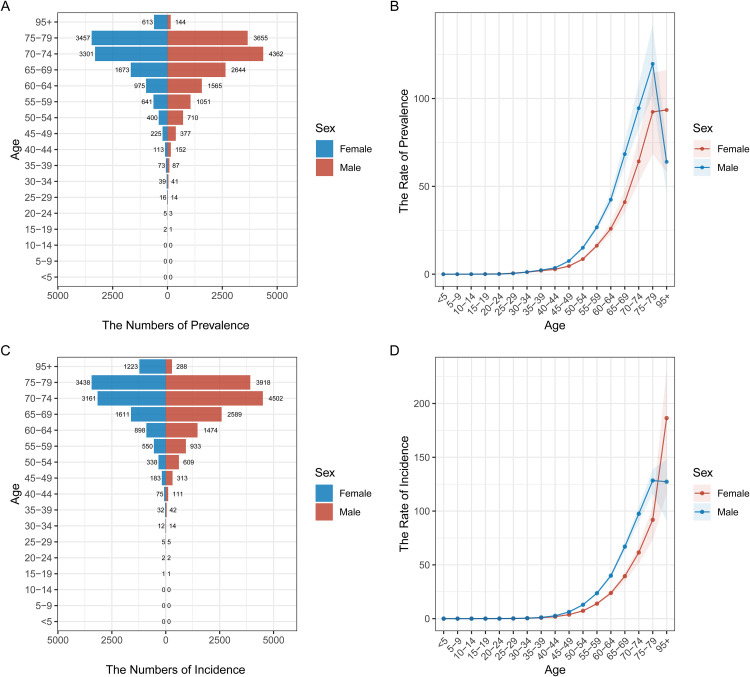
Age and Gender Distribution of Pancreatic Cancer Prevalence and Incidence in Japan (1990–2021). (A) **Prevalence by age and gender**: Japan has the highest prevalence in males aged 75 and older, while females show lower numbers. (B) **Rate of prevalence by age group**: The rate increases significantly after 65 years, with a higher prevalence in males compared to females. (C) **Incidence by age and gender**: Similar to prevalence, incidence is higher in males, particularly among the elderly. (D) **Rate of incidence by age group**: There is a sharp rise in incidence with age, with males showing higher rates, particularly beyond 70 years.

**Fig 5:**
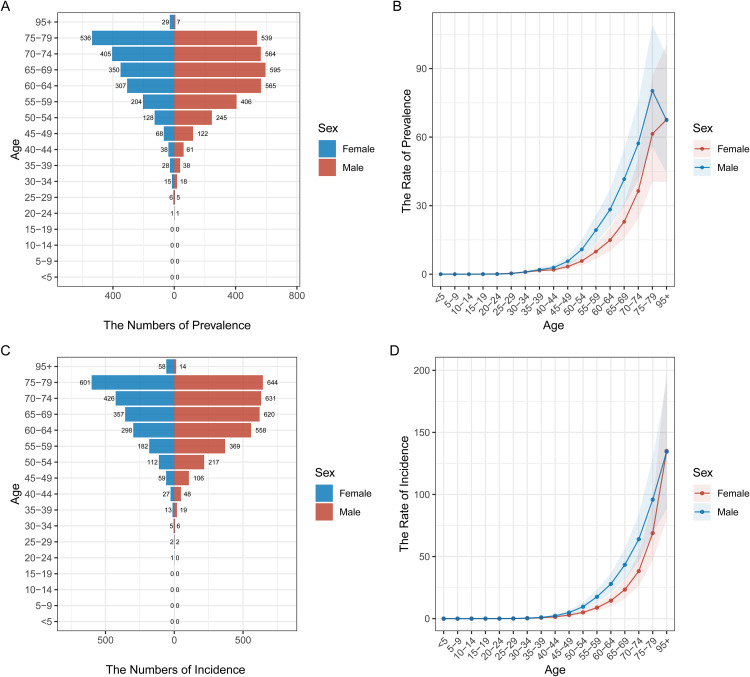
Age and Gender Distribution of Pancreatic Cancer Prevalence and Incidence in Korea (1990–2021). (A) **Prevalence by age and gender**: Males consistently show higher prevalence rates compared to females, particularly in the age group 70-79 years. (B) **Rate of prevalence by age group**: Prevalence rates increase steadily with age, with higher rates observed in males. (C) **Incidence by age and gender**: The incidence of pancreatic cancer is also higher in males, with increasing trends noted among older individuals. (D) **Rate of incidence by age group**: Incidence rates follow a similar age-dependent increase, with a higher burden observed in males.

**Fig 6:**
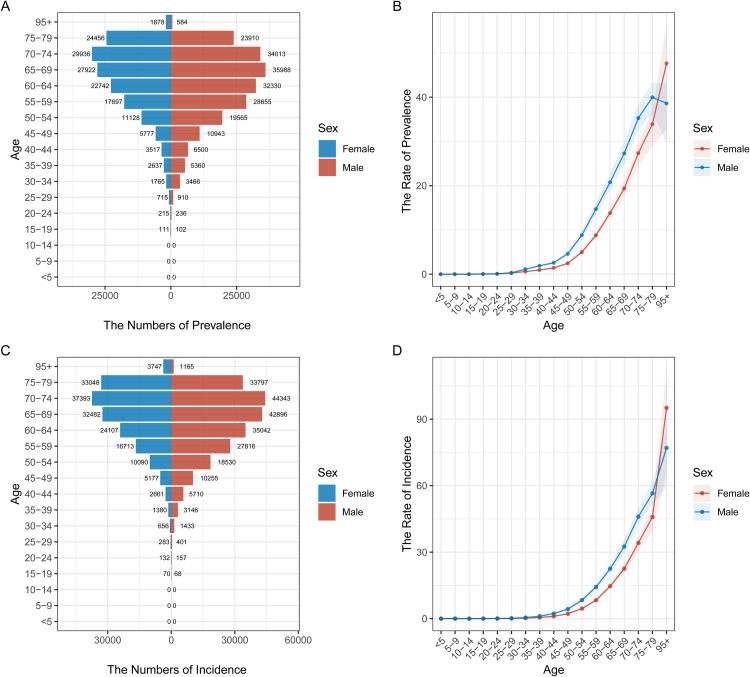
Global Age and Gender Distribution of Pancreatic Cancer Prevalence and Incidence (1990–2021). (A) **Prevalence by age and gender**: Globally, males have a higher prevalence of pancreatic cancer, particularly in individuals aged 65 and older. (B) **Rate of prevalence by age group**: The rate of prevalence escalates with age, with males consistently having higher rates across all age groups. (C) **Incidence by age and gender**: The global incidence of pancreatic cancer is higher in males, with sharp increases noted after age 65. (D) Rate **of incidence by age group**: Incidence rates increase with age, with the highest rates observed among individuals over 75 years.

**Fig 7:**
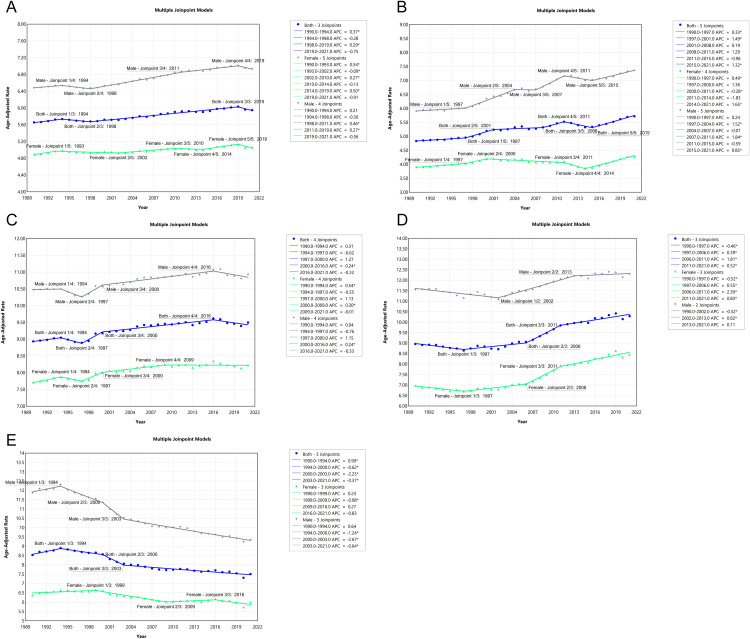
Joinpoint Regression Analysis of Age-Standardized Pancreatic Cancer Death Rates (1990–2021). (A) **Global trends**: Mortality rates have increased for both males and females, with a steeper rise among males, particularly from 1993 to 2019. (B) **China**: A significant increase in mortality rates is observed, particularly among males, starting from 2015. (C) **USA**: Mortality rates for males show a steady rise, while females have experienced a slower increase. (D) **Japan**: Mortality rates showed a slight decline initially, but began to increase significantly after 2000, particularly among males, with a more pronounced rise after 2006.**(E) Korea**: A notable decline in mortality rates has been observed in recent years, particularly after 2015, suggesting improvements in healthcare interventions.

Figs 2 to 6 illustrate the age and gender distribution of pancreatic cancer prevalence and incidence across different regions, including China (Fig 2), the USA (Fig 3), Japan (Fig 4), Korea (Fig 5), and global data (Fig 6). Across all regions, a clear trend emerges: pancreatic cancer prevalence and incidence increase sharply with age, particularly in individuals aged 65 and older. For all regions, men consistently show higher numbers of both prevalence and incidence compared to women, especially in the older age groups (75 + years). In China, the USA, Japan, and Korea, males dominate in both prevalence and incidence rates across all age groups, with the highest rates observed among those over 75 years old. Globally (Fig 7), the trends are consistent with the regional patterns, showing that the prevalence and incidence of pancreatic cancer escalate significantly with age, and men experience a heavier burden of the disease compared to women.

### Trends in age-standardized pancreatic cancer deaths and incidence using joinpoint analysis

**Fig 8:**
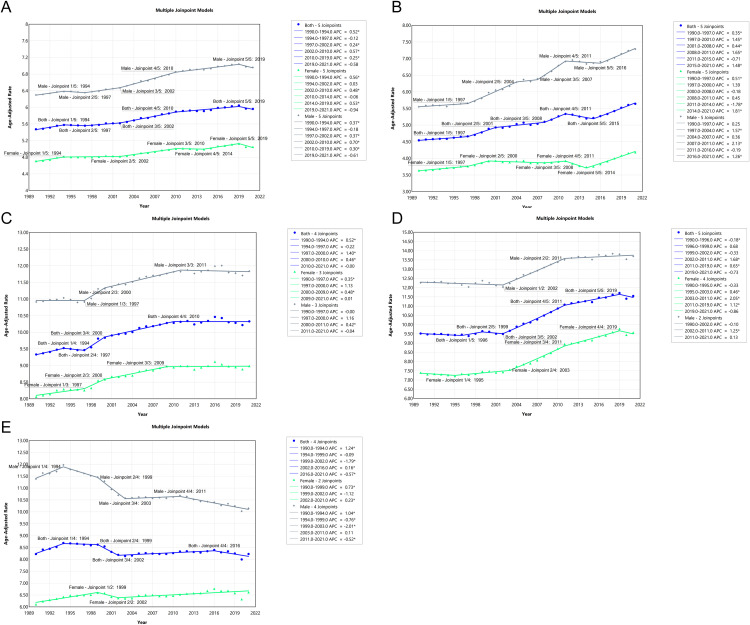
Joinpoint Regression Analysis of Age-Standardized Pancreatic Cancer Incidence Rates (1990–2021). (A) **Global trends**: The incidence of pancreatic cancer has been steadily increasing for both genders, with males showing a steeper rise. (B) **China**: A steep increase in incidence is noted among males, with a slower increase for females. (C) **USA**: Both males and females show a steady rise in incidence, with males showing higher rates. (D) **Japan**: The incidence rate remained relatively stable in the early years of the study period, but a noticeable increase occurred from the mid-2000s, especially in males, with a more pronounced rise post-2010. (E) Korea: The incidence rate for males increased steadily from 1990 to 1994, peaked around 1994, and then declined until 2003. From 2003 to 2011, the rate remained relatively stable, entering a plateau phase, followed by a decline after 2011. For females, the incidence rate increased from 1990 to 1999, peaked around 1999, and then declined until 2002, with a modest increase noted thereafter.

Figs 7 and 8 present joinpoint regression models that analyze the trends in age-standardized pancreatic cancer deaths (Fig 7) and incidence (Fig 8) from 1990 to 2021 in various regions, including global data, China, USA, Japan, and Korea. The joinpoint models indicate periods of significant change in trends for both mortality and incidence, allowing for the identification of inflection points where the rates of increase or decrease shifted. In **Fig 7**, which highlights death trends, **Panel A** shows global data where both men and women exhibit increasing death rates, with men experiencing sharper increases, particularly from 1993 to 2019. **Panel B** shows China’s mortality trend, where men experienced a significant rise in mortality rates, particularly after 2015, while women’s mortality also increased, but at a slower pace compared to men. **Panel C** depicts the USA, where a steady increase in male mortality is observed, while female death rates rise at a slower rate. In Japan, the mortality rates showed a slight decline initially but began to increase significantly after 2000, especially among males, with a more pronounced rise from 2006 onward, as shown in **Panel D**. Meanwhile, in Korea, **Panel E** indicates a significant decline in death rates in recent years. **Fig 8** focuses on incidence trends. **Panel A** reveals a steady global increase in incidence for both sexes, though the rate of increase for males is more pronounced. **Panel B** shows China’s incidence trends, highlighting a steep rise in male incidence, while females experience a gradual increase. **Panel C** illustrates that incidence in the USA has risen steadily for both genders. In Japan, the incidence rate remained relatively stable in the early years of the study period. However, a noticeable increase occurred from the mid-2000s, especially in males, with a more pronounced rise post-2010, as shown in **Panel D**. In contrast, **Panel E** highlights Korea’s more stable incidence trends. For males, the incidence rate increased steadily from 1990 to 1994, peaked around 1994, and then declined until 2003. From 2003 to 2011, the rate remained relatively stable, entering a plateau phase, followed by a decline after 2011. For females, the incidence rate increased from 1990 to 1999, peaked around 1999, and then declined until 2002, with a modest increase noted thereafter.

### Age-period-cohort analysis for pancreatic cancer deaths, incidence, and prevalence

**Fig 9:**
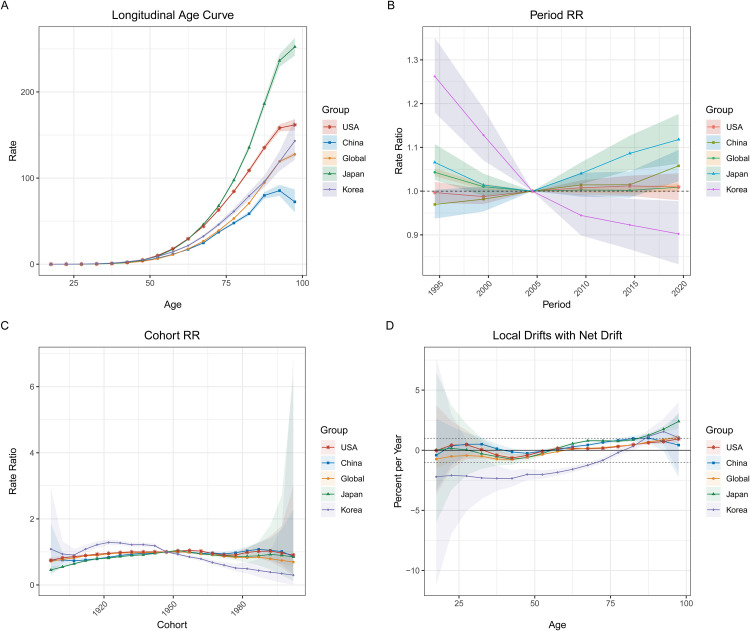
Age-Period-Cohort Analysis of Pancreatic Cancer Mortality (1990–2021). (A) **Longitudinal age curve**: Mortality rates increase exponentially with age, particularly after age 60, in all regions. (B) Period **effects**: A marked rise in relative risk is observed after 2000, particularly in China and globally. (C) **Cohort effects**: More recent birth cohorts (post-1950) show an increased risk of pancreatic cancer, particularly in China. (D) **Local drifts with net drift**: Mortality rates rise consistently with age, with the steepest increases in the oldest age groups (75+), especially in China.

**Fig 10:**
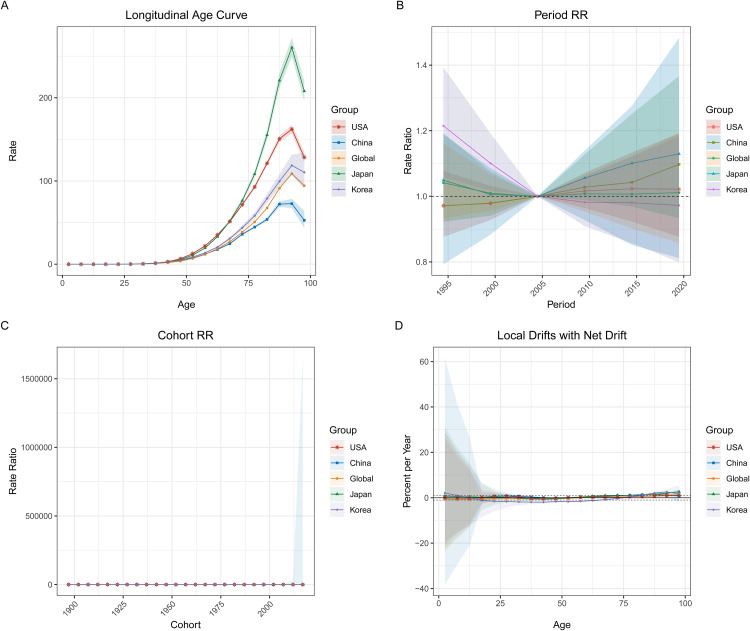
Age-Period-Cohort Analysis of Pancreatic Cancer Incidence (1990–2021). This figure presents the Age-Period-Cohort (APC) analysis of pancreatic cancer incidence trends in the USA, China, Japan, Korea, and globally. (A) **Longitudinal age curve**: Incidence rates increase sharply with age, particularly after age 65, in all regions. The steepest rise is observed in Japan and the USA. (B) **Period effects**: Period rate ratios (RR) indicate an increased risk for pancreatic cancer incidence after 2000, with China showing the highest increase. (C) **Cohort effects**: The cohort risk ratio shows a consistent increase in incidence risk for more recent birth cohorts (post-1950), especially in China and globally. (D) Local **drifts with net drift**: Local drift analysis demonstrates a steady increase in incidence across all age groups, with older populations (65+) showing the most significant rise.

**Fig 11:**
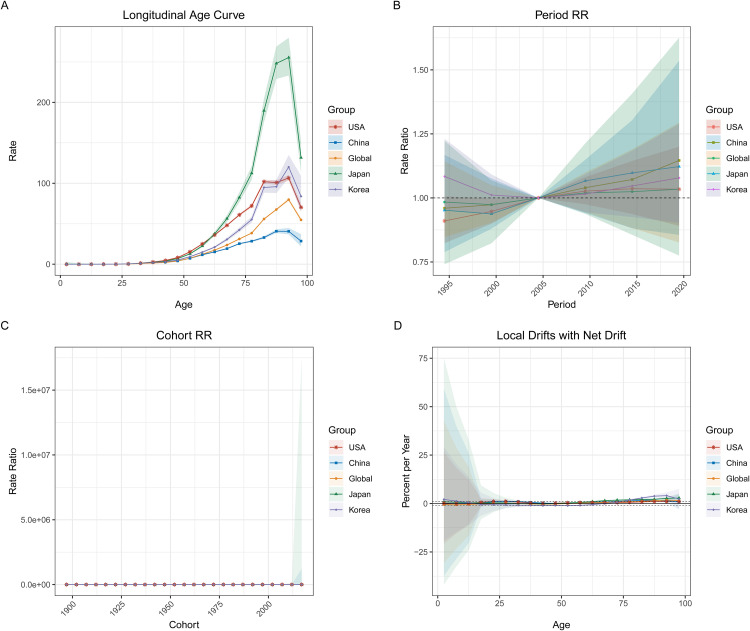
Age-Period-Cohort Analysis of Pancreatic Cancer Prevalence (1990–2021). This figure illustrates APC analysis for pancreatic cancer prevalence across different regions, including the USA, China, Japan, Korea, and globally. (A) **Longitudinal age curve**: Prevalence rates increase with age, particularly among individuals aged 65 years and older, in all regions. (B) **Period effects**: Period rate ratios indicate an upward trend in pancreatic cancer prevalence risk after the year 2000, especially in China and globally. (C) **Cohort effects**: Prevalence risk increases with newer birth cohorts, with a noticeable rise in cohort risk ratio for populations born after 1950. (D) **Local drifts with net drift**: Local drifts indicate a steady rise in prevalence, with a pronounced effect among older individuals, highlighting the age-related burden of pancreatic cancer.

Figs 9 to 11 illustrate the APC analysis for pancreatic cancer deaths, incidence, and prevalence across global regions, including China, Korea, Japan, and the USA. This analysis breaks down the trends into age effects (longitudinal age curves), period effects (period rate ratios), and cohort effects (cohort rate ratios), providing insights into the temporal dynamics of pancreatic cancer across different age groups, periods, and birth cohorts. In **Fig 9**, the **longitudinal age curve (Panel A)** reveals that pancreatic cancer mortality rates increase exponentially with age, particularly after 60 years old, across all regions. **Period effects (Panel B)** suggest a notable rise in relative risk (RR) after 2000 in China and globally, while **cohort effects (Panel C)** demonstrate a consistent rise in risk for recent birth cohorts (after 1950), particularly in China and globally. **Local drifts (Panel D)** show minor increases in death rates across most age groups, with the steepest rises seen in those over 75. **Fig 10** focuses on **incidence rates**, with the **age curve (Panel A)** showing an increasing trend for all regions, particularly after age 65, with China and the USA exhibiting the sharpest rise. **Period effects (Panel B)** again indicate rising risks post-2000 for China and the global cohort, while **cohort effects (Panel C)** follow a similar trend, with higher risks for more recent birth cohorts. **Local drifts (Panel D)** for incidence reflect a consistent upward trend across all age groups, with more pronounced increases in older age categories. In **Fig 11**, the **prevalence data** reveal similar trends, with rising rates among older age groups (Panel A) and increasing risk across periods, particularly in China (Panel B). **Cohort effects (Panel C)** indicate higher prevalence in newer birth cohorts, while **local drifts (Panel D)** demonstrate a steady rise in prevalence, particularly in those aged 65 and older.

### Predicted trends in pancreatic cancer ASIR, ASMR, ASPR, and DALYs (2020–2030)

**Fig 12:**
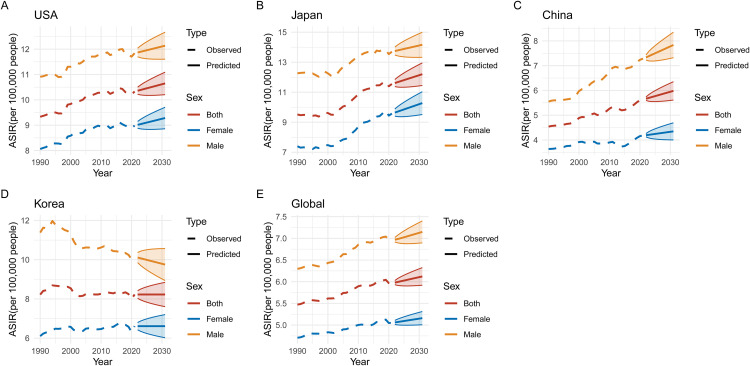
Predicted Trends in Age-Standardized Incidence Rates (ASIR) of Pancreatic Cancer (2020–2030). This figure depicts the projected age-standardized incidence rates (ASIR) for pancreatic cancer from 2020 to 2030 in the USA, Japan, China, Korea, and globally. (A-E) Each panel represents a different region (USA, Japan, China, Korea, and global data). Predictions indicate an increase in ASIR for both sexes in most regions, particularly Japan and the USA, with males consistently having higher predicted rates than females. In Korea, ASIR is expected to remain relatively stable, while China and global rates are projected to increase moderately.

**Fig 13:**
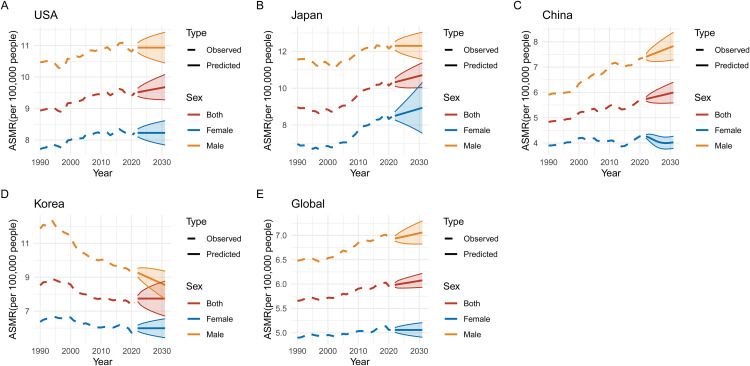
Predicted Trends in Age-Standardized Mortality Rates (ASMR) of Pancreatic Cancer (2020–2030). This figure illustrates predicted age-standardized mortality rates (ASMR) of pancreatic cancer across five regions for the period 2020–2030. (A-E) Each panel shows ASMR predictions for the USA, Japan, China, Korea, and globally. Japan and the USA are projected to exhibit the highest mortality rates, particularly for males. In contrast, Korea is expected to show stable or declining trends in ASMR, while China and global mortality rates are likely to increase, highlighting the public health challenge posed by pancreatic cancer.

**Fig 14:**
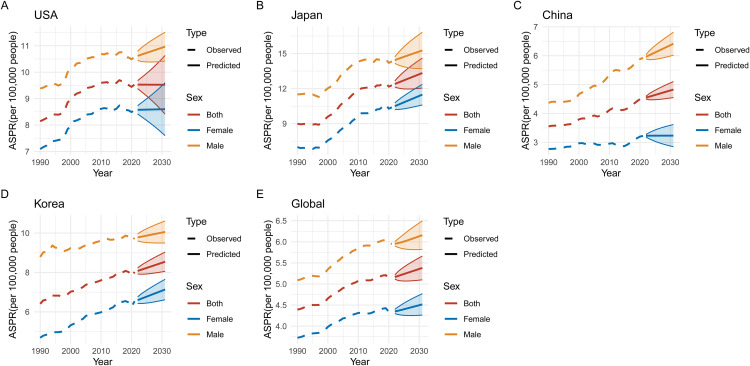
Predicted Trends in Age-Standardized Prevalence Rates (ASPR) of Pancreatic Cancer (2020–2030). This figure presents predicted trends for age-standardized prevalence rates (ASPR) of pancreatic cancer from 2020 to 2030 across the USA, Japan, China, Korea, and globally. (A-E) The highest prevalence is projected for Japan and the USA, with ASPR expected to rise significantly for males. China and Korea are predicted to show more stable trends, while global prevalence is anticipated to increase moderately, underscoring the need for targeted prevention strategies.

**Fig 15:**
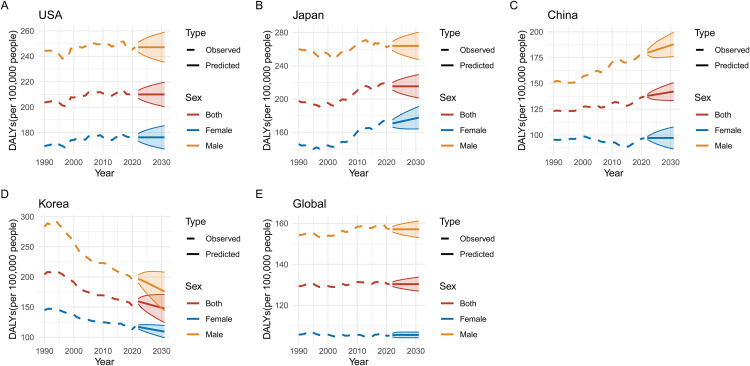
Predicted Trends in Disability-Adjusted Life Years (DALYs) Due to Pancreatic Cancer (2020–2030). This figure shows the predicted trends in Disability-Adjusted Life Years (DALYs) due to pancreatic cancer from 2020 to 2030 in the USA, Japan, China, Korea, and globally. (A-E) DALYs are projected to increase most significantly in Japan and the USA, reflecting the growing burden of pancreatic cancer, particularly among males. Conversely, Korea’s DALYs are expected to decline, suggesting successful interventions. China’s DALYs are projected to continue rising, emphasizing the need for improved healthcare resources and early detection methods. Global DALYs indicate a steady increase, with males predicted to bear a larger burden compared to females.

Figs 1215 illustrate the predicted trends for pancreatic cancer in ASIR, ASMR, age-standardized prevalence rate (ASPR), and DALYs across five regions: USA, Japan, China, Korea, and global data from 2020 to 2030. The projections highlight significant differences between regions and sexes, with marked increases expected in some areas. In **Fig 12**, ASIR projections show that Japan and the USA are expected to experience the highest incidence rates by 2030, with ASIR increasing for both sexes but consistently higher among males in all regions. **Fig 13** predicts that ASMR will follow a similar pattern, with Japan continuing to show the highest predicted mortality rates, while Korea’s rates appear more stable. **Fig 14** focuses on ASPR, where the highest projected prevalence rates are also found in Japan, followed closely by the USA, with male prevalence higher in every region. Finally, **Fig 15** predicts that DALYs will rise the most in Japan and the USA, reflecting the overall burden of the disease, while Korea is expected to have the lowest DALYs by 2030.

## Discussion

This study provides important insights into the trends and future projections of pancreatic cancer burden across China, Korea, Japan, and the USA from 1990 to 2021. Our analysis reveals that Japan and the USA will face the highest pancreatic cancer incidence and mortality rates by 2030, with males consistently showing higher rates compared to females in all countries. While Korea exhibited relatively stable trends with a slight decrease in mortality rates, China experienced a notable rise in both incidence and DALYs, particularly among males, signaling a growing public health concern. These findings suggest that, although there are differences in the rate of increase, pancreatic cancer continues to be a significant health burden in all four countries, especially as populations age. The use of the GBD 2021 data allowed for detailed cross-country comparisons, emphasizing the need for targeted public health interventions, improved early detection strategies, and resource allocation to address this increasing disease burden. The projections up to 2030 further highlight the urgency for country-specific approaches to mitigate future impacts, especially in high-risk populations such as older males.

The findings of this study align with previous research on the global burden of pancreatic cancer, particularly the growing incidence and mortality rates in high-income countries such as Japan and the USA. Studies using GBD data have consistently highlighted the rising trend of pancreatic cancer in these regions, driven by aging populations and the prevalence of major risk factors such as smoking, obesity, and diabetes [14,17–19]. For example, some studies have reported that the projected pancreatic cancer burden in Japan and the USA is among the highest globally, which corresponds to the results of our study predicting continued increases in ASIR and ASMR in these countries through 2030 [20,21]. Similar trends have been noted in other studies, particularly in East Asia and North USA, where the combination of an aging demographic and lifestyle changes has led to a substantial increase in pancreatic cancer rates [13,21].

On the other hand, some studies have suggested a potential plateau in pancreatic cancer incidence in countries with advanced healthcare systems due to improvements in screening and early detection programs [1,8,22,23]. However, our findings differ from these studies, as the predicted incidence rates in Japan and the USA continue to rise, particularly among older males. This discrepancy could reflect the limitations of current screening methods in detecting early-stage pancreatic cancer or the continued impact of risk factors such as smoking and high body mass index (BMI), which are more prevalent in males [13,20,24]. Moreover, our findings on the gender disparity in pancreatic cancer trends, with males exhibiting consistently higher incidence and mortality rates, are supported by previous research that has linked these differences to higher exposure to carcinogenic risk factors among men [24–27].

In this study, several advanced statistical techniques were employed to assess pancreatic cancer trends, including joinpoint regression and APC modeling. The joinpoint regression analysis provided a nuanced view of the annual percent change in pancreatic cancer incidence, mortality, and DALYs, allowing the identification of significant inflection points where the rates of change shifted markedly [16]. This method proved particularly useful in capturing the trends in countries like Japan and the USA, where recent advances in healthcare have led to noticeable reductions in mortality. The APC model further supports these findings by highlighting the significant impact of cohort effects and aging on pancreatic cancer risks, particularly in older males, with rising incidence and mortality rates in both Japan and the USA. This aligns with the increasing trends observed after 2000. Additionally, the APC analysis dissected the temporal effects of aging, periods, and birth cohorts, illustrating how generational lifestyle changes and medical advancements influence pancreatic cancer risks [28–31]. The findings from the APC model provide additional insight by highlighting cohort effects, especially in China, where younger generations are showing a higher relative risk compared to previous cohorts. This is in line with the observed trends in the Joinpoint regression analysis, where male mortality rates in China significantly increased after 2015, and cohort effects were particularly influential in shaping the burden. The study’s findings revealed that while aging remains a dominant factor, cohort effects also significantly contributed to the increased burden of pancreatic cancer in China, where younger generations are showing a higher relative risk compared to previous cohorts. This aligns with the results from the Joinpoint regression analysis, which showed a sharp rise in male mortality rates in China after 2015, indicating the impact of younger cohorts and generational shifts on pancreatic cancer trends. Predictive modeling extended the analysis to 2030, providing future projections of incidence, mortality, and DALYs, which underscore the urgency of targeted interventions in high-risk populations.

Despite the valuable insights provided by this study, several limitations should be considered. First, the study relies heavily on data from the GBD database, which, while comprehensive, is derived from various sources that may vary in quality and completeness, particularly in low-income countries where healthcare data are often underreported or misclassified [17,32,33]. Additionally, the predictive models used in this analysis are based on the assumption that current trends in demographics, healthcare access, and treatment will remain unchanged, potentially overlooking future healthcare innovations or interventions that could alter the course of pancreatic cancer incidence and mortality [20,34]. Furthermore, while the joinpoint and APC models were instrumental in identifying inflection points and temporal trends, these models do not fully account for the complex interplay of genetic, environmental, and behavioral factors that contribute to pancreatic cancer risk. The study also did not incorporate the potential impact of emerging screening technologies, such as liquid biopsies or AI-driven diagnostic tools, which could significantly affect early detection rates in the future. Moving forward, it is recommended that future research include real-time data from cancer registries and national healthcare systems to improve the accuracy of estimates. Moreover, greater attention should be paid to the genetic and molecular mechanisms of pancreatic cancer across different populations, which could explain regional disparities in outcomes. Further studies should also explore the role of novel technologies, such as AI-driven diagnostics and personalized medicine, in improving early detection and treatment strategies. Lastly, longitudinal studies examining lifestyle factors and their relationship to pancreatic cancer incidence would provide valuable insights into prevention strategies, particularly for aging populations.

In conclusion, this study underscores the significant and growing burden of pancreatic cancer, particularly in aging populations. Future research should focus on improving early detection methods, utilizing emerging technologies like artificial intelligence and molecular diagnostics, and addressing genetic and environmental factors contributing to regional disparities. High-quality real-time data from cancer registries will be essential to refining disease burden estimates. Additionally, prevention strategies targeting lifestyle factors, such as smoking, obesity, and diabetes, must be prioritized. International collaboration will be critical in advancing research and developing global interventions to reduce the impact of pancreatic cancer. By addressing these areas, future efforts can better manage and mitigate the rising challenges posed by this highly aggressive disease.

### Highlights

- Comparative analysis of pancreatic cancer trends across China, Korea, Japan, and the USA.
- Projected increase in pancreatic cancer burden in Japan and the USA by 2030.
- Joinpoint and age-period cohort models are used for trend analysis and future predictions.
- Rising incidence in aging populations emphasizes the need for targeted public health measures.
- Predictive models highlight the urgency for improved early detection and treatment strategies.

## Abbreviations

ASIR: Age-Standardized Incidence Rate
ASMR: Age-Standardized Mortality Rate
DALYs: Disability-Adjusted Life Years
APC: Age-Period-Cohort
GBD: Global Burden of Disease
IHME: Institute for Health Metrics and Evaluation
